# Identification of novel bladder sensory GPCRs

**DOI:** 10.14814/phy2.14840

**Published:** 2021-05-01

**Authors:** Tilmira A. Smith, Brittni N. Moore, Andres Matoso, Dan E. Berkowitz, Jennifer J. DeBerry, Jennifer L. Pluznick

**Affiliations:** ^1^ Department of Physiology Johns Hopkins University Baltimore MD USA; ^2^ Wayne State University School of Medicine Detriot MI USA; ^3^ Departments of Pathology, Urology, and Oncology Johns Hopkins University Baltimore MD USA; ^4^ Department of Anesthesiology and Perioperative Medicine University of Alabama at Birmingham Birmingham AL USA

**Keywords:** ectopic, olfactory receptor, opsin, taste receptor

## Abstract

Sensory GPCRs such as olfactory receptors (ORs), taste receptors (TRs), and opsins (OPNs) are now known to play important physiological roles beyond their traditional sensory organs. Here, we systematically investigate the expression of sensory GPCRs in the urinary bladder for the first time. We find that the murine bladder expresses 16 ORs, 7 TRs, and 3 OPNs. We additionally explore the ectopic expression of these GPCRs in tissues beyond the bladder, as well as the localization within the bladder. In future work, understanding the functional roles of these bladder sensory GPCRs may shed light on novel mechanisms which modulate bladder function in health and disease.

## Graphical abstract

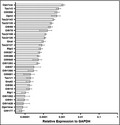

## 1 INTRODUCTION

Olfactory receptors (ORs), taste receptors (TRs), and photoreceptors/opsins (OPNs) have been primarily recognized for their key roles in smell, taste, and sight. However, recent studies have highlighted novel roles for these receptors in a variety of additional processes, including modulation of renal, cardiovascular, and pulmonary function (Dalesio et al., 2018). The great majority of TRs, and all OPNs and ORs, are G‐protein‐coupled receptors (GPCRs). In fact, ORs represent the largest family of GPCRs, with ~1000 receptors in mice and ~350 receptors in humans (Godfrey et al., (2004); Malnic et al., 2004). TRs belong to one of two GPCR families: type 1 (Tas1r) for sweet/umami taste, and type 2 (Tas2r) for bitter taste. ORs and TRs respond to chemical ligands, whereas OPNs are activated by photons. In addition to the visual photoreceptors, there are also non‐visual photoreceptors that have been recently discovered: Opn3, Opn4, and Opn5.

The roles of these sensory GPCRs have been explored in a number of tissues beyond the eyes, nose, and tongue. For example, OR1D2 guides sperm to the egg during the fertilization process (Spehr et al., 2003), sweet TRs expressed in mouse pancreatic islets induce insulin secretion *in vitro* (Nakagawa et al., (2009)), and Opn4 mediates blood vessel dilation (Sikka et al., 2014). The bladder is well known to express classical GPCRs, such as the β adrenergic receptors, which can respond to external stimuli to modulate urothelium function (Otsuka et al., 2008). Although other studies have reported the presence sensory GPCRs in bladder (Elliott et al., (2011); Uhlen et al., 2015; Weber et al., 2018; Yu et al., 2019), there has not yet been a systematic exploration of sensory GPCRs in the bladder. In this study, our goal was to systemically screen the bladder for ORs, TRs, and OPNs, in the hopes of beginning to uncover and understand the localization and function of sensory GPCRs in the bladder.

## 2 METHODS

### 2.1 Animals

All animal protocols were approved by the Johns Hopkins University Animal Care and Use Committee. All mice used in this study were C57Bl/6J and were fed ab libitium and housed on a 14–10 light–dark cycle.

### 2.2 Taqman screen on bladder

C57BL/6J mice (two males, two females, 3 months of age) were euthanized in the morning between 10 a.m. and 12 p.m. by CO_2_ inhalation. Whole bladders were harvested and homogenized in TRIzol reagent (Thermofisher 15596018), and RNA was extracted. RNA was then further processed using the Qiagen RNeasy Mini Kit with DNase digest protocol per manufacturer's instructions. Fragment analysis was performed to ensure good RNA quality. Isolated RNA was reverse transcribed using the High Capacity cDNA Reverse Transcription Kit (Thermofisher 4368813). Quantitative PCR screening was performed using custom Taqman Low Density Array (TLDA) cards containing the probes noted in Table 1 (probes marked by an asterisk in Table 1 were not used in follow‐up experiments because we found that the negative controls were not as reliable for these probes; instead, we used Opn1sw Mm01135620_g1, Olfr558 Mm01279850_m1, Opn3 custom probe APU66GD, Olfr78 Mm00628115_m1). For the TLDA card, ORs were selected based on the 40 most highly expressed human ORs as reported by Flegel et al. (2013). Of these 40 human ORs: 5 genes had no murine ortholog (NCBI) and thus were not included, two genes had two murine orthologs listed and these were both included, and one gene had a murine ortholog that was annotated as a pseudogene but it was included as it contains both a start and a stop site. In addition, we included 5 genes which our lab had previously observed as ectopically expressed (Olfr1392, Olfr1393, Olfr691, Olfr693, Olfr99, and Olfr31 (Pluznick et al., 2009; Rajkumar et al., 2014)), for a total of 43 ORs. The card additionally included all 35 murine bitter taste receptors as well as the taste receptors for umami and sweet, five opsin genes (including all three non‐visual opsins), four G proteins associated with sensory signaling (Gnal, Gnat3, GNAT1, GNAT2), receptor transporting proteins 1–3, and GAPDH. One TLDA card was used per bladder sample. About 1 µg of bladder cDNA was added to each fill port (8 µg cDNA in total per card; ~21 ng per reaction). Cycling was performed using Taqman Fast Advanced Mastermix (Thermofisher 4444556).

**TABLE 1:** List of genes and probes included on the TLDA card

	Gene Name	Assay ID	Reference Sequence
G Proteins			
1	Gnal	Mm01258217_m1	NM_010307.3;NM_177137.5
2	Gnat3	Mm01165313_m1	NM_001081143.1
3	GNAT1	Hs00181100_m1	NM_000172.3;NM_144499.2
4	GNAT2	Hs00292542_m1	XM_011541264.2;NM_005272.3
Olfactory Receptors (Olfr)			
1*	Olfr78	Mm00453733_s1	NM_001168503.1;NM_130866.4
2	Olfr322	Mm03040519_sH	NM_207693.1
3	Olfr732	Mm01322515_s1	NM_146665.2
4*	Olfr558	Mm00530250_s1	NM_147093.3
5	Olfr435	Mm00730406_s1	NM_146653.1
6	Olfr13	Mm00455512_s1	NM_146652.1
7	OLFR658	Oc04252848_gH	NM_001171477.1
8	Olfr211	Mm00528861_s1	NM_146912.1
9	Olfr177	Mm00838183_sH	NM_146996.2
10	Olfr410	Mm00837649_s1	NM_146707.1
11	Olfr56	Mm01294180_s1	NM_010999.2
12	Olfr90	Mm01279749_s1	NM_146477.2
13	Olfr1352	Mm01173416_s1	NM_147071.2
14	Olfr166	Mm04211699_sH	NM_147068.1
15	Olfr418	Mm01294388_s1	NM_146651.2
16	Olfr355	Mm00730208_s1	NM_146625.1
17	Olfr314	Mm01353840_s1	NM_001011760.2
18	Olfr91	Mm00731552_s1	NM_182714.2
19	Olfr287	Mm01308734_s1	NM_001011780.1
20	Olfr288	Mm04214148_s1	NM_001011733.2
21	Olfr411	Mm00527201_s1	NM_146709.2
22	Olfr618	Mm00529874_s1	NM_147047.2
23	Olfr273	Mm00528191_s1	NM_146824.1
24	Olfr267	Mm00528927_s1	NM_146920.2
25	Olfr71	Mm00450916_s1	NM_019486.1
26	Olfr15	Mm00435446_s1	NM_008762.2
27	Olfr26	Mm02344389_s1	NM_146783.2
28	Olfr933	Mm00732053_s1	NM_146441.1
29	Olfr646	Mm01280303_s1	NM_147056.1
30	Olfr57	Mm00730842_s1	NM_147041.2
31	Olfr714	Mm01610019_s1	NM_147033.2
32	Olfr11	Mm03012560_s1	NM_146542.2
33	Olfr873	Mm01175052_s1	NM_146561.1
34	Olfr308	Mm00730170_s1	NM_146621.1
35	Olfr935	Mm00847580_s1	NM_146746.1
36	Olfr1366	Mm00525728_s1	NM_146283.2
37	Olfr1428	Mm00730594_s1	NM_146678.2
38	Olfr1392	Mm00836974_s1	NM_146470.2
39	Olfr1393;Olfr10	Mm00848749_g1	NM_146471.1;NM_206822.1
40	Olfr691	Mm00529987_s1	NM_147061.1
41	Olfr693	Mm00729330_s1	NM_146453.2
42	Olfr99	Mm01280705_s1	NM_146515.2
43	Olfr31	Mm01314539_s1	NM_147027.2
44	Olfr545	Mm01279086_s1	NM_146840.1
Opsins (Opn)			
1	Opn4	Mm00443523_m1	NM_001128599.1;NM_013887.2
2*	Opn1sw	Mm00432058_m1	NM_007538.3
3	Opn5	Mm00710998_m1	NM_181753.4
4	Opn1mw	Mm00433560_m1	NM_008106.2
5*	Opn3	Mm00438648_m1	NM_010098.3
Accessory Proteins			
1	Rtp1	Mm01619091_m1	NM_001004151.2
2	Rtp3	Mm00462169_m1	NM_153100.2
3	Rtp2	Mm02374643_s1	NM_001008230.3
Sweet/Umami Taste Receptors (Tas1r)			
1	Tas1r1	Mm00473433_m1	NM_031867.2
2	Tas1r2	Mm00499716_m1	NM_031873.1
3	Tas1r3	Mm00473459_g1	NM_031872.2
Bitter Taste Receptors (Tas2r)			
1	Tas2r120	Mm03014488_s1	NM_207023.1
2	Tas2r109	Mm01161497_g1	NM_207017.1
3	Tas2r137	Mm01167554_s1	NM_001025385.1
4	Tas2r124	Mm01161473_s1	NM_207026.1
5	Tas2r108	Mm00498514_s1	NM_020502.1
6	Tas2r143	Mm01700139_s1	NM_001001452.1
7	Tas2r110	Mm01160274_s1	NM_199155.2
8	Tas2r135	Mm01701729_s1	NM_199159.1
9	Tas2r116	Mm01160271_s1	NM_053212.1
10	Tas2r129	Mm03014501_s1	NM_207029.1
11	Tas2r134	Mm01701728_s1	NM_199158.1
12	Tas2r126	Mm01702063_s1	NM_207028.1
13	Tas2r131	Mm01702072_s1	NM_207030.1
14	Tas2r105	Mm00498502_s1	NM_020501.1
15	Tas2r102	Mm03014393_s1	NM_199153.2
16	Tas2r104	Mm01702013_s1	NM_207011.1
17	Tas2r140	Mm03011269_s1	NM_021562.1
18	Tas2r125	Mm01160225_s1	NM_207027.1
19	Tas2r119	Mm00498529_s1	NM_020503.2
20	Tas2r136	Mm00663741_s1	NM_181276.1
21	Tas2r114	Mm01702033_s1	NM_207019.1
22	Tas2r106	Mm01702023_s1	NM_207016.1
23	Tas2r139	Mm00663740_s1	NM_181275.1
24	Tas2r118	Mm01702043_s1	NM_207022.1
25	Tas2r115	Mm01160239_s1	NM_207020.1
26	Tas2r103	Mm01161465_s1	NM_053211.1
27	Tas2r144	Mm01700149_s1	NM_001001453.1
28	Tas2r130	Mm01701719_s1	NM_199156.1
29	Tas2r113	Mm01702024_m1	NM_207018.1
30	Tas2r121	Mm01702053_s1	NM_207024.1
31	Tas2r123	Mm01167370_s1	NM_207025.1
32	Tas2r107	Mm01701709_s1	NM_199154.1
33	Tas2r122	Mm03039326_s1	NM_001039128.1
34	Tas2r117	Mm04213039_s1	NM_207021.1
35	Tas2r138	Mm01700131_s1	NM_001001451.1
Control			
	Gapdh	Mm99999915_g1	NM_001289726.1

### 2.3 qRT‐PCR for other organs

Three C57BL/6J male mice (4 months of age) were anesthetized with 10 mg/ml sodium pentobarbital and perfused with 1X PBS to remove blood. Organs collected were brain, colon, eye, heart, kidney, skeletal muscle, and tongue. RNA extraction was performed as described above, and reverse transcription was performed using the Qiagen Quantitect Reverse Transcription Kit (Qiagen cat# 205311). The top 10 sensory receptors detected in the bladder, along with negative control receptors which were not detected in the bladder, were screened via quantitative PCR using the Taqman Gene Expression Mastermix (Thermofisher 4369016). Mock reactions (no Reverse Transcriptase enzyme) were run as controls: 5 ng cDNA was used per reaction.

### 2.4 Microdissection of mouse bladder and qRT‐PCR

C57BL/6J mice (four males, four females, 3 months of age) were anesthetized with 10 mg/ml sodium pentobarbital and perfused with collagenase to ease separation of bladder layers. Whole bladders were harvested and cut in half, longitudinally. One half was used as the whole bladder control. The other half was microdissected in 1X PBS under a dissecting microscope. Fine forceps were used to peel off the urothelium. The remaining tissue was considered as the muscle layer. The tissues were homogenized in RLT buffer, and RNA was extracted following instructions from the Qiagen RNeasy Micro Kit (Qiagen cat# 74004). Quantitative PCR was performed using probes for luminal and muscle layers, as well as for the top 10 sensory receptors detected in the TLDA cards, following the same protocol mentioned above. Mock controls (no Reverse Transcriptase enzyme) were also performed for each probe. 5 ng cDNA was used per reaction.

### 2.5 Statistics

All data are represented as mean ± standard deviation (SD). Statistical analyses were performed using Prism 8.2.1 by one‐way ANOVA (multiple comparisons/tukey). A value of *p* < 0.05 considered as significant. Because this is an exploratory study, p‐values are descriptive rather than hypothesis testing.

## 3 RESULTS

### 3.1 Sensory GPCRs are found in murine bladder

**FIGURE 1:**
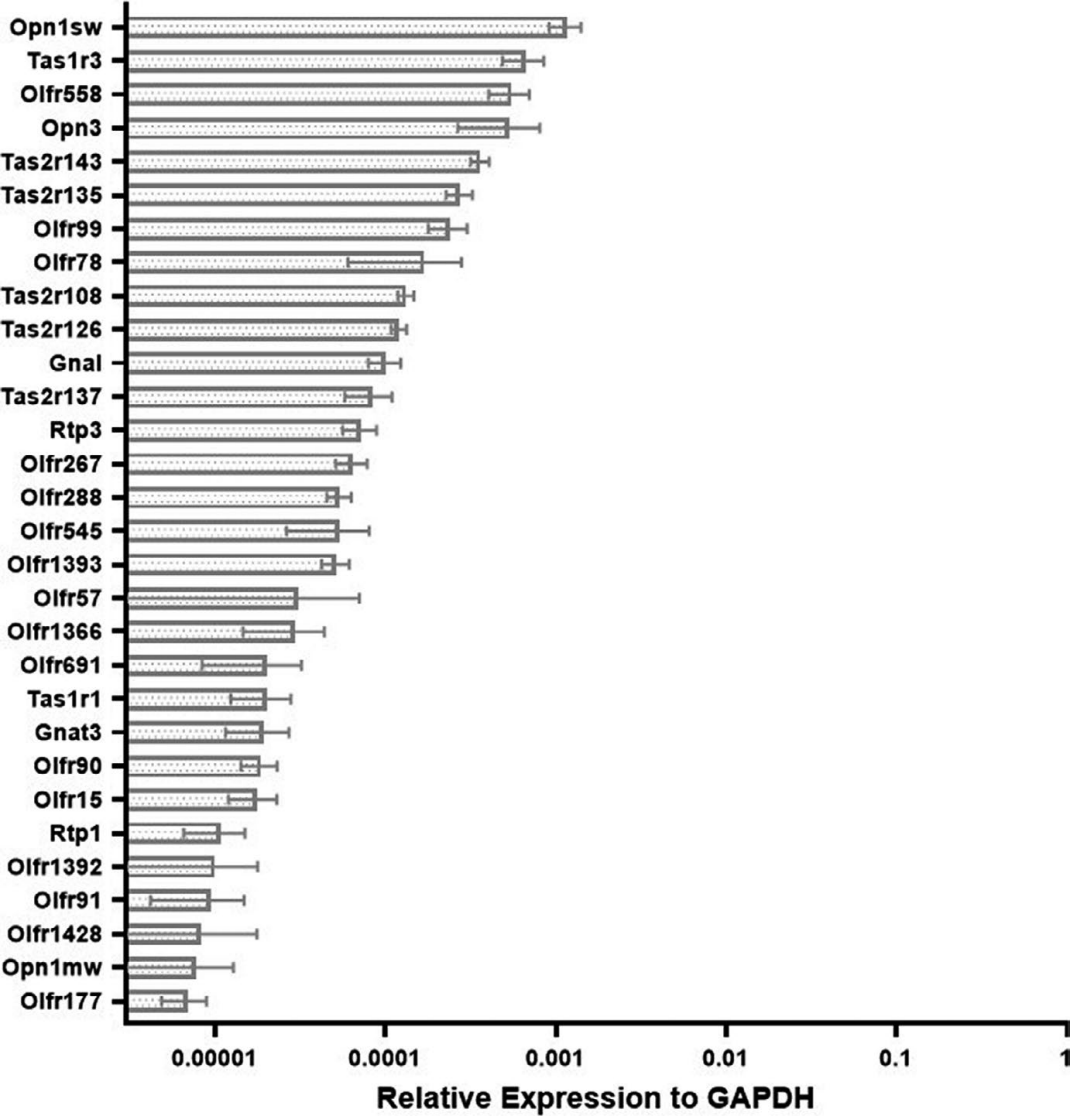
Novel G‐protein coupled sensory receptors in murine bladder. mRNA expression of detected transcripts (30/94 receptors screened) in murine bladder was quantified by qRT‐PCR using Taqman low density array cards. Data are represented as mean ± SD. N = 4. SD for Olfr57 = ±3.93E‐05, Olfr1393 = ±7.78E‐06, Olfr1428 = ±9.38E‐06, Opn1mw = ±5.11E‐06

**FIGURE 2:**
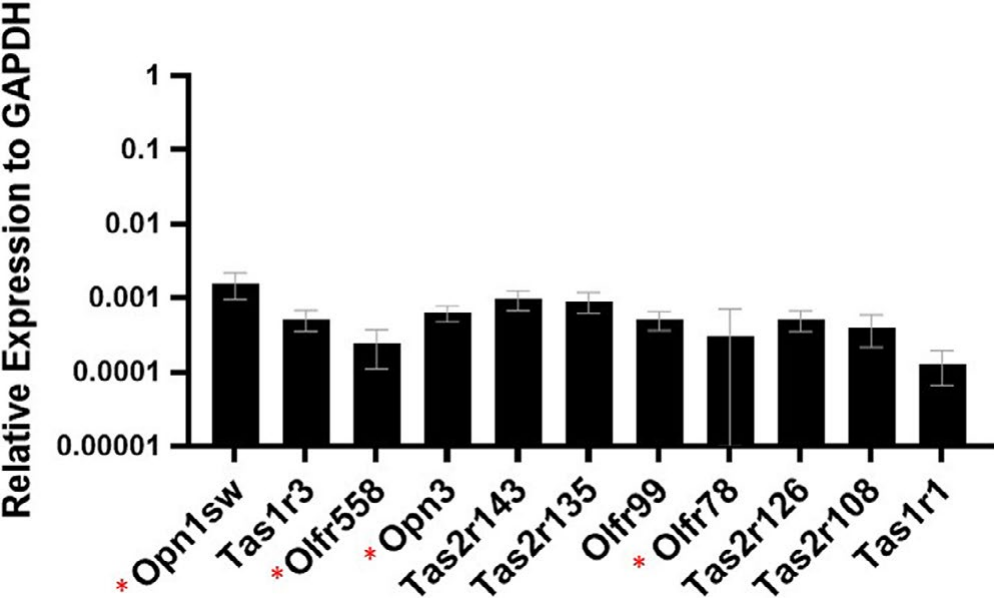
Confirming expression of top sensory GPCRs. Expression of the top 10 sensory receptors was confirmed following the screening using the same bladder samples. * a different probe used than in the initial TaqMan screen. Data are represented as average ± SD. N = 4. SD for Olfr78 = ±6.19E‐04

Custom Taqman Low Density Array (TLDA) cards were used to screen reverse‐transcribed RNA from C57BL/6J bladders (two males, two females). Probes for the genes on each card are listed in Table 1 and described in the Methods, and include sensory GPCRs and accessory proteins. Of the 94 transcripts screened, 30 were detected in the bladder (Ct<36). In Figure 1, sensory receptors are arranged in descending order of expression in the bladder, relative to GAPDH. The top 10 transcripts (Ct<31) include two OPNs, three ORs, four bitter TRs, one sweet/umami TR, and the G protein for olfaction (G_olf_). The gene for the blue cone receptor, Opn1sw, had the highest expression on all four array cards. The sweet/umami TR, Tas1r3, had the second highest expression. Two short‐chain fatty acid receptors that our lab has previously studied in the kidney, Olfr558 (Halperin Kuhns et al., 2019) and Olfr78 (Pluznick et al., 2013), were also detected. Although this screen was not powered to examine sex differences, there were no clear patterns of differences by sex. We subsequently confirmed the expression of the top 10 sensory GPCRs in a separate experiment (Figure 2). In future experiments, we focused on the receptors with the highest level of expression in the bladder.

### 3.2 Sensory GPCRs are found in various organs

**FIGURE 3:**
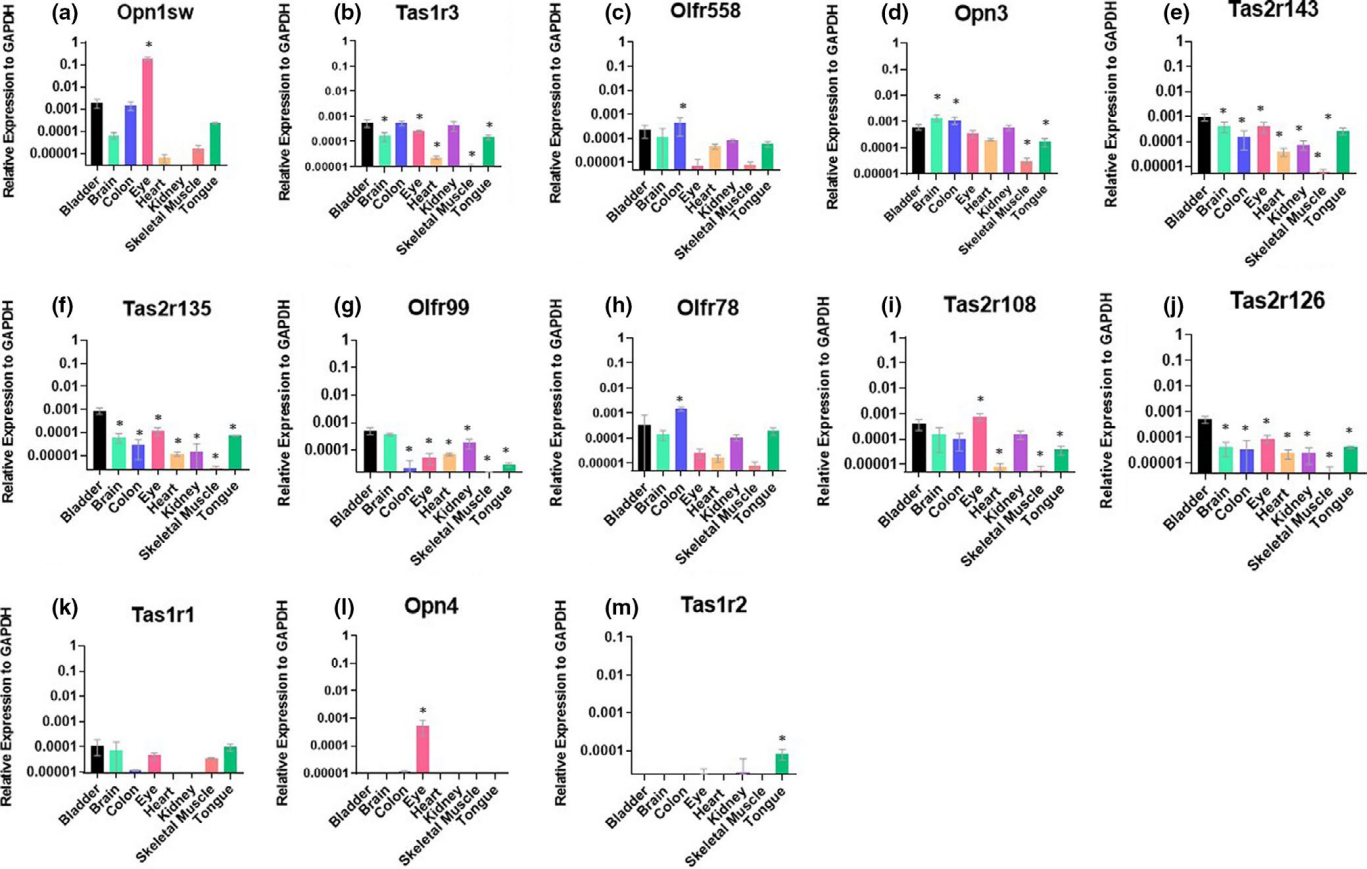
Screening of top bladder sensory GPCRs in various organs. mRNA expression of top bladder sensory receptors was assessed in various tissues and quantified by qRT‐PCR. Data are represented as mean ± SD. N = 3–8. * *p* < 0.05 for bladder vs. the indicated tissue by one‐way ANOVA. SD for Olfr558 brain = ±1.36E‐04, Tas2r135 kidney = ±1.78E‐05, Olf78 bladder = ±4.9E‐04, Tas2r126 colon = ±3.99E‐05, Tas1r1 brain = ±8.49E‐05

To determine if the sensory receptors expressed in the bladder are specific to the bladder, or, if they are broadly expressed, we performed a tissue qPCR screen. This screen included brain, colon, eye, heart, kidney, skeletal muscle, and tongue. We screened the top 10 bladder sensory receptors from the TLDA cards: Opn1sw, Tas1r3, Olfr558, Opn3, Tas2r143, Tas2r135, Olfr99, Olfr78, Tas2r108, and Tas2r126 (Figure 3a‐j). Tas1r1 was also included because it was the only other type 1 taste receptor detected in bladder (Figure 3k), and type 1 taste receptors function as dimers. Also included in this screen were two negative controls that were not detected in bladder: Opn4 and Tas1r2. Opn4 and Tas1r2 were also negative in the other tissues screened, with the exception of their respective positive control tissues: Opn4 was only detected in the eye, and Tas1r2 was detected only in the tongue (Figure 3l‐m).

Of the sensory receptors found to be expressed in the bladder, all were expressed in a variety of tissues in addition to the bladder (and the eye or tongue). However, the distribution of each individual receptor was different. For example, Opn1sw had the highest expression in the eye, and had varied levels of expression in most other tissues, but was absent from the kidney. Tas1r3 was detected in nearly every tissue, but had little to no expression in skeletal muscle. By contrast, Tas2r143, Tas2r135, and Tas2r126 (Figure 3e,f,j) had higher expression in the bladder than in the other tissues examined. The highest Olfr78 expression was found in the colon, whereas the highest Olfr99 expression was found in the bladder and brain. Collectively, these data show that it is not uncommon to find a subset of sensory receptors outside of traditional sensory organs, with varying levels of expression.

### 3.3 Localization of top bladder sensory GPCRs within murine bladder

**FIGURE 4:**
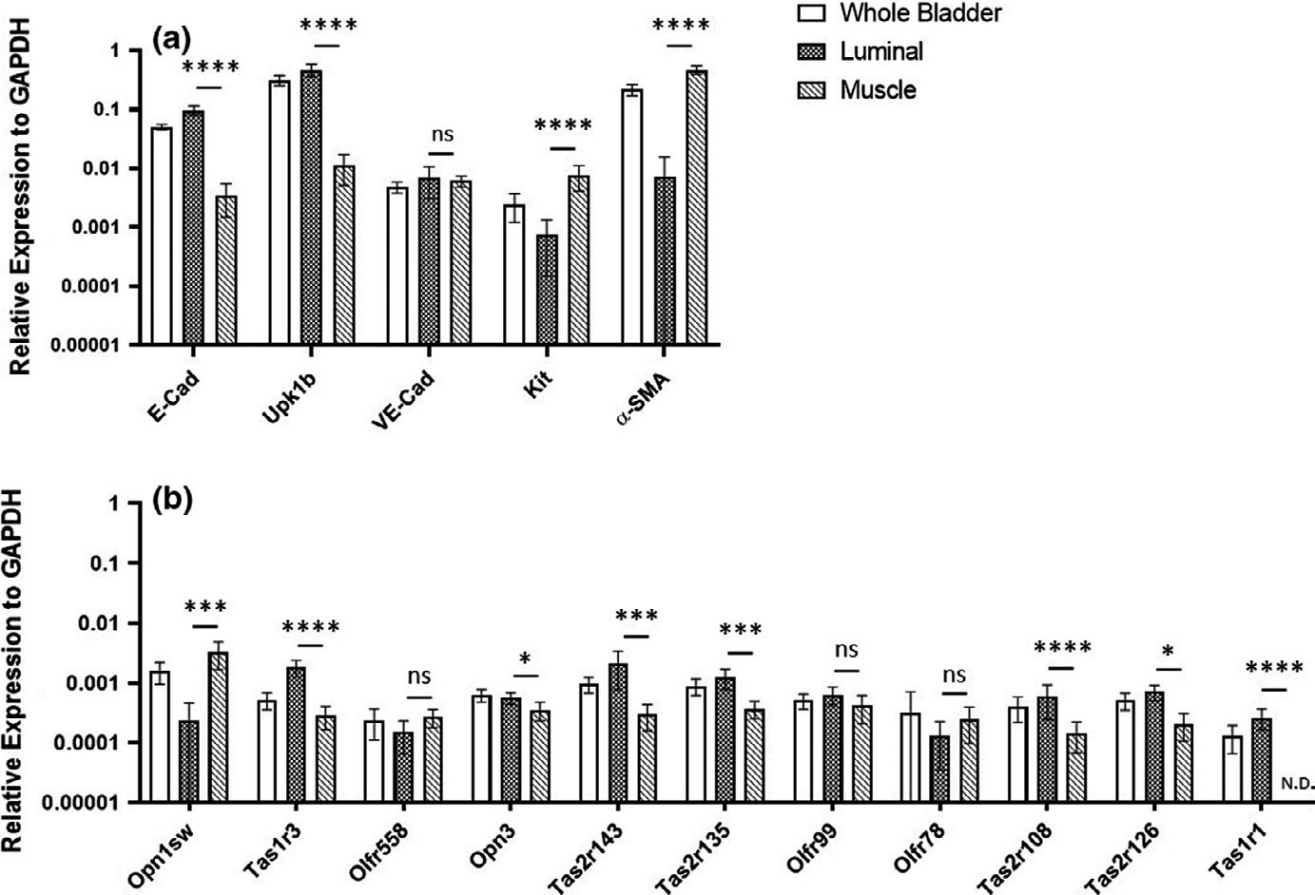
Localization of top 10 sensory GPCRs in enriched bladder tissue populations. Bladders were harvested from mice and microdissected into a luminal fraction and a muscle fraction. Enriched tissue populations were confirmed using known markers of luminal and muscle fractions (a) and mRNA expression of top receptors in whole bladder, luminal, and muscle fractions were assessed (b) by qRT‐PCR. Data are represented as mean ±SD. N=6–8. *p < 0.05, **p < 0.01, ***p < 0.001, ****p < 0.0001 by one‐way ANOVA. ns = not significant. N.D. = not detected. SD for SMA luminal = ±8.35E‐03, Opn1sw luminal = ±2.25E‐04, Olfr78 whole bladder = ±3.97E‐04

To determine the localization of the top bladder sensory receptors within the bladder, qPCR was performed on enriched bladder cell populations. Bladders were harvested from 8 C57BL/6J mice; half of the bladder served as the whole bladder control, whereas the other half was microdissected into the luminal fraction and the muscle fraction. First, qPCR was performed using probes for genes which we expected to be enriched in the luminal (epithelial Cadherin/ E‐Cad and Uroplakin 1B/ Upk1b) or muscle layers (C‐Kit and Alpha Smooth Muscle Actin/ α‐SMA). These data confirmed that our microdissection protocol had significantly enriched each fraction as expected (Figure 4a); vascular Endothelial Cadherin/VE‐Cad is an endothelial marker (endothelial cells are found in the suburothelium between the urothelium and muscle layers).

Next, we performed qPCR on these enriched cell fractions for the top 10 sensory receptors from the TLDA cards, along with Tas1r1 (Figure 4b). We found Opn1sw to be the only receptor significantly enriched in the muscle fraction, although Olfr558 and Olfr78 trended toward higher expression in the muscle fraction. Olfr99 also showed no significant enrichment in either fraction. Tas1r3, Opn3, Tas2r143, Tas2r135, Tas2r108, Tas2r126, and Tas1r1 were significantly enriched in the luminal fraction. We did not observe any sex differences.

**FIGURE 5:**
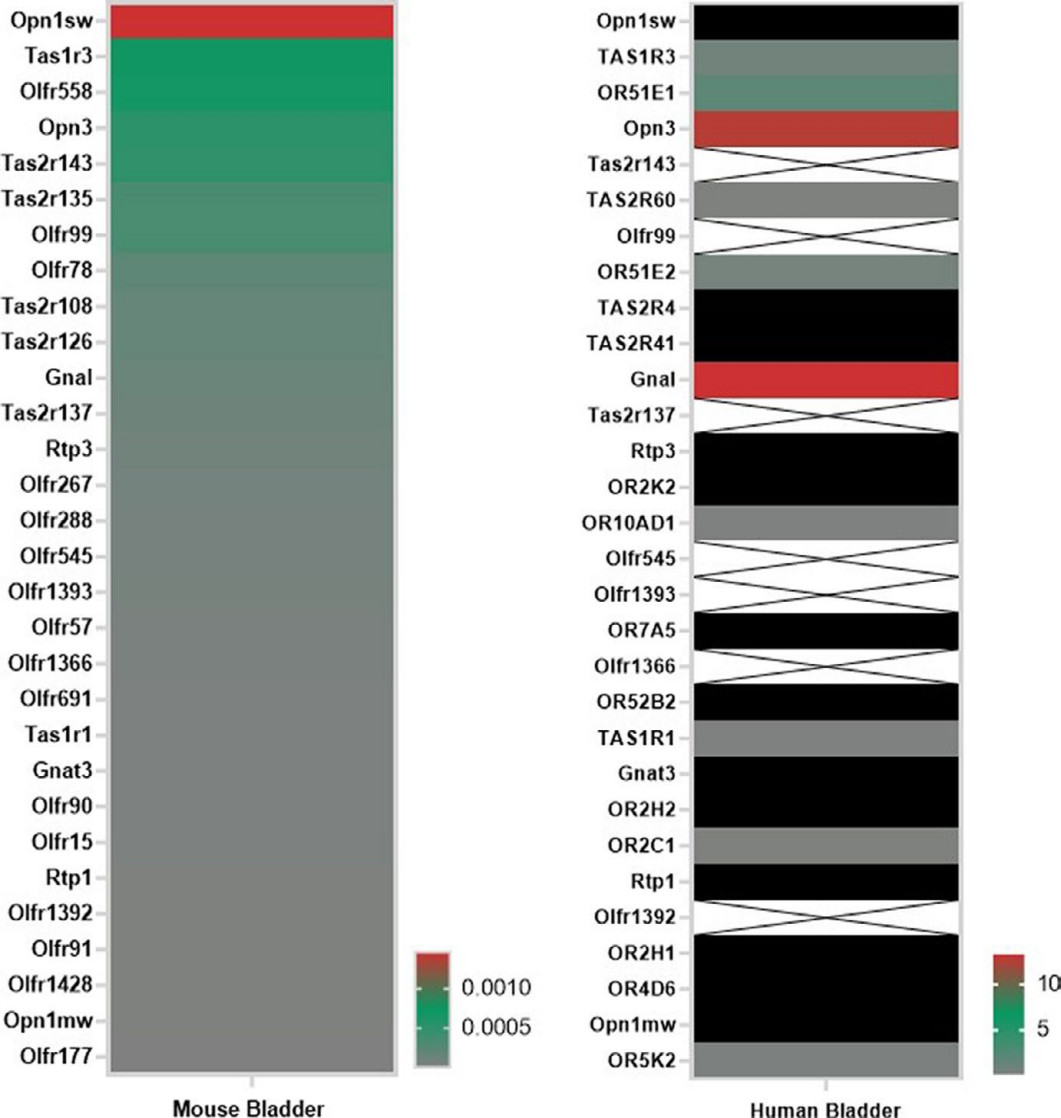
A comparison of sensory GPCRs in mouse and human bladder. Using the top 30 sensory GPCRs detected in our Taqman array on mouse bladder, we compared this qPCR data (left) with the RNAseq data from Uhlen et al. (2015) on human bladder (right). On the right, the black color was used to indicate an expression level equal to zero; the X’s were used to indicate that there was no human ortholog for the corresponding mouse gene. Please note that for the ORs and bitter TRs (Tas2r), the human gene name differs from the murine gene name

Additionally, we compared the top 30 sensory GPCRs from our study against RNASeq data from *human* bladder (Uhlen et al., 2015) (Figure 5), and noted a few receptors that were expressed in both human and murine bladder including: Tas1r3, Olfr558/OR51E1, Opn3, Tas2r135/TAS2R60, and Olfr78/OR51E2. Among these, Opn3 had the highest expression in human bladder.

## 4 DISCUSSION

In this study, we systemically examined the expression of sensory GPCRs in the bladder for the first time. We found that of the 94 transcripts screened, 30 were expressed in the bladder. The most highly expressed transcripts included two OPNs, three ORs, and five TRs, and these GPCRs exhibited a widespread but varied expression in other tissues. Within the bladder, most of these GPCRs were enriched in the luminal fraction.

### 4.1 Other reports of sensory GPCRs in bladder

Although ours is the first systemic examination of sensory receptors in the bladder, there are other reports which have examined individual TRs or ORs in the bladder. For example, Elliott, et al reported that sweet TRs (Tas1r2 and Tas1r3 dimer) are expressed in human and rat urothelium on the protein level, and suggested they may play a role in bladder contraction (Elliott et al., 2011). While our study also saw expression of Tas1r3 in murine bladder, we instead observed co‐expression of Tas1r1 (Tas1r1 and Tas1r3 would constitute the umami TR). Localization of Tas1r3 expression to the rat urothelium by Elliott, et al. is in agreement with our data showing significant enrichment of Tas1r3 in the luminal fraction of mouse bladder. The differences between our findings may reflect species differences, or the different methodologies used. In a separate study, Zhai, et al. reported that 19 bitter TRs were detected in mouse detrusor muscle (Zhai et al., 2016). However, the bitter TRs with the highest expression in this study were not detected in our screen, and the bitter TRs found in our screen were found at relatively low levels in their study. Notably, we screened whole bladder, whereas Zhai et al screened detrusor muscle, and this may account for some of these differences – especially given that we see enrichment of bitter TRs in the luminal fraction. Finally, we are aware of one study which reported expression of an OR in human bladder (with upregulated expression in bladder cancer): OR10H1 (Weber et al., 2018). This OR is not evolutionarily conserved (Niimura et al., 2014) and thus its murine orthologs are uncertain. However, the putative mouse orthologs, Olfr239 and Olfr55, were not included in our screen and have not been reported in mouse bladder. While we performed qPCR to collect data from mouse tissue only, there is a study that performed RNA sequencing on mouse and human bladder tissue (Yu et al., 2019). In comparison to our data, Yu and others only detected the expression of three sensory GPCRs (Opn1sw, Opn3, Tas1r3) in mouse bladder and three sensory GPCRs in human bladder (Opn3, OR51E1/Olfr558, and OR51E2/Olfr78). Notably, this single‐cell sequencing data also indicate that Opn3 may be more highly expressed in human bladder as compared with mouse. Uhlen et al. (2015) performed RNA sequencing on several *human* tissues including bladder and detected the expression of the following sensory GPCRs that were also detected in our screen: Tas1r3, Olfr558/OR51E1, Opn3, Tas2r135/TAS2r60, Olfr78/OR51E2, Gnal, Tas2r137, Olfr288/OR10AD1, Tas1r1, Olfr15/OR2C1, and Olfr177/OR5K2. Though not bladder specifically, other tissues within close proximity, such as the urethra, have been shown to also express sensory GPCRs, specifically bitter TRs (Kummer & Deckmann, 2017).

In our study, we report that Opn1sw and Opn3 are expressed in the murine bladder. It is not yet known how these opsins are activated *in vivo*: does light naturally reach Opn1sw or Opn3 in the bladder? We know that the short‐wave photoreceptor, Opn1sw, is activated by wavelengths in the blue light range. A study in 2018 demonstrated that blue light induces vasorelaxation via Opn3 and Opn4, implying that Opn3 is indeed activated by blue light *in vivo*. However, we should note that while most groups report that blue light activates Opn3 (Barreto Ortiz et al., 2018; Nayak et al., 2020; Regazzetti et al., 2018), whereas at least one group reports that it may not (Ozdeslik et al., 2019). Another possibility is that these opsins may have a light‐independent role (Leung & Montell, 2017) – it would be intriguing to explore whether some opsins may also be activated by a chemical ligand, or by mechanotransduction. Moving forward, it will be key to understand the mechanism of activation for opsins in the bladder.

Although we do not yet have enough data to speculate on the functional role of specific sensory GPCRs in the bladder, we can draw from the existing literature of sensory GPCRs in other tissues to intuit potential function roles. For example, several studies have shown that some sensory GPCRs (including Olfr78 (Pluznick et al., 2013) and Olfr558 (Halperin Kuhns et al., 2019), both of which are found in murine bladder) are activated by bacterial metabolites. Thus, it is tempting to speculate that these receptors may be activated to modulate bladder contraction or pain mechanisms in conditions such as cystitis. In future studies, it will be critical to examine the functional role of each of these novel sensory GPCRs, and to understand how signaling of these GPCRs may influence bladder function in health and disease.

### 4.2 Limitations

There are several limitations to our study. First, our analysis is focused on RNA, and RNA expression is not necessarily indicative of functional protein. In future studies, it will be important to look at both protein and at function. Conversely, a relatively low level of expression on the RNA level does not necessarily mean that the GPCR does not play a key role in bladder function: for example, if a GPCR is expressed only in a minority cell type, then relative abundance by whole bladder PCR would be low. Thus, GPCRs which are found at low or even absent levels of expression may still play significant roles in bladder function. Finally, we acknowledge that there may be species differences in expression of these sensory GPCRs, and this will be important to examine going forward.

## CONFLICT OF INTEREST

The authors declare that they have no conflict of interest.

## AUTHOR CONTRIBUTION

T.A.S., D.E.B., and J.L.P. conceived these studies. T.A.S. and B.N.M. performed experiments. T.A.S., B.N.M., A.M., D.E.B., J.J.D., and J.L.P. analyzed data. T.A.S. and J.L.P. wrote the manuscript; T.A.S., B.N.M., A.M., D.E.B., J.J.D., and J.L.P. edited and approved the manuscript.
